# Cascaded domain engineering optical phased array for 2D beam steering

**DOI:** 10.1515/nanoph-2023-0382

**Published:** 2023-10-20

**Authors:** Jingwei Li, Huaibin Zheng, Yuchen He, Yanyan Liu, Xiaoyong Wei, Zhuo Xu

**Affiliations:** Electronic Materials Research Laboratory, Key Laboratory of the Ministry of Education and International Center for Dielectric Research, School of Electronic and Information Engineering, Xi’an Jiaotong University, Xi’an, 710049, Shaanxi, China; National Key Laboratory of Electromagnetic Space Security, The 53rd Research Institute of China Electronics Technology Group Corporation, 300308, Tianjin, China

**Keywords:** domain engineering, optical phased array, beam steering

## Abstract

The current approach to 2D optical phased array (OPA) encounters challenges, such as the requirement for a highly tunable laser that is incompatible with certain 2D beam-steering applications or significant power consumption, large antenna spacing and complex wiring resulting from independent control of array elements. To address these challenges, we propose an OPA architecture based on cascaded periodically poled LiNbO_3_ sequences, a multi-layered domains engineered structure within the LiNbO_3_ electro-optic crystal, only two control electronics to program the 2D beam-steering trajectory with a range of approximately *θ*_*y*_ = ±20° and *θ*_*z*_ = ±16° through simulations. This structure enables the uniform distribution of phase differences between adjacent array elements (adjacent domains) upon beam exit from the crystal, ensuring optimal performance. The aim of this study is to develop a methodology that employs domain engineering techniques for designing high-performance phase-controlled devices with customized functional units and sequences in electro-optical crystals. Our research has implications for emerging optoelectronic applications, such as customizable optical interconnects and integrated LiDAR systems.

## 1 Introduction

Reorienting light at high speeds plays a crucial role in numerous applications [1, 2], including modern telecommunications networks [3, 4], biomedical imaging [5, 6], light detection and ranging (LiDAR) [7–9], and wireless optical communications [10]. Optical phased arrays (OPAs) have the ability to manipulate the wavefront of light and achieve non-mechanical beam steering by controlling the phase of each emitter. They offer a promising alternative to traditional laser beam steering units that rely on moving mirrors and lenses in optical sensing and free-space communication modules [11–13]. The emerging implementation of OPA in the form of photonic integrated circuits (PIC) has the ability to achieve beam steering in a compact, robust, and low-energy consumption manner.

Two-dimensional optical beam steering is typically achieved through two main methods: utilizing a 1-D array of grating-based optical antennas, where the formed beam can be steered in one dimension by adjusting the relative phase between the array elements and in the other dimension through wavelength tuning [14–18], or employing a 2-D phased array, where beam-steering in both dimensions is accomplished via relative phase adjustments between the array elements [8, 11, 19]. Despite its excellent performance, the former method requires a highly tunable laser source and is not compatible with certain applications such as optical communication [20, 21]. Additionally, it typically achieves a smaller steering range through wavelength tuning compared to the relative phase adjustments between the OPA elements. To realize a narrow beam with wide field-of-view steering capability through another method, an OPA consisting of numerous closely spaced array elements within a large aperture is necessary. For instance, in a conventional 2-D OPA with an *N* × *N* array of elements, N^2^ phase tuning control electronics are necessary for per-element phase adjustment. Designing such a 2-D OPA that can produce complex far-field patterns through controlled electronics presents a significant challenge, especially when dealing with a large number of array elements. This challenge includes not only the daunting task of establishing hundreds or even thousands of electrical connections but also exponentially growing complexity involved in propelling the control system to accommodate the increased number of array elements [22]. Cascading strategy, originally proposed by Thomas and Fainman, offers a good solution to this problem [23]. It achieves full scan control by using a minimum number of control electronics. The favorable outcomes of this experiment have engendered a pervasive embrace of cascading strategy as a viable approach to mitigate the burden of control units in OPA [24–26].

In our previous research, we introduced a structure for one-dimensional beam steering called a cascaded periodically poled electro-optical phased array [27]. This structure consists of multiple layers of periodically poled cascaded structures, which are engineered within the electro-optic crystal using domain engineering techniques. The distinctive design ensures uniform phase distribution among adjacent array elements (adjacent ferroelectric domains) as the beam exits the crystal. The operating principle of OPA involves the independent control of each array element’s phase to achieve their function. However, this poses a significant challenge in terms of critical trade-offs among the number of components, control electronics, and power consumption. The cascaded periodically poled electro-optical phased array overcomes these trade-offs by enabling manipulation of all phase tuning units through a single control electronics unit. This renders it an outstanding contender for realizing photonic integrated circuit beam steering components. Our previous work has demonstrated the efficacy of cascaded periodically poled electro-optical phased arrays for one-dimensional beam steering. The pursuit of a 2-D beam steering system that excels in precision, speed, resolution, and integration, has posed a significant challenge.

Here, we present a 2D OPA architecture based on cascaded periodically poled electro-optical technology. Our design achieves 2D beam steering without the need for a laser wavelength tuning component, while featuring centralized control of all array elements through just two electronic signals, regardless of the number of elements. This approach presents the possibility of increased integration and reduced power consumption. The novel 2D OPA architecture consists of two cascaded periodically poled structures, which are engineered within a LiNbO_3_ crystal utilizing domain engineering techniques. The proposed 2D OPA architecture was utilized to demonstrate two-dimensional beam steering with a range of approximately *θ*_*y*_ = ±20° and *θ*_*z*_ = ±16°. The path of the beam steering can be programmed through only two control electronics. The objective of this investigation is to examine the influence of cascaded periodically poled structures on phase control in emitted beams within LiNbO_3_ crystals, as well as the underlying mechanisms of regulation. In detail, our aim is to clarify the physical mechanism that connects cascaded periodically poled sequences with the macroscopic performance of phase-controlled devices, and establish a correlation between functional units (ferroelectric domains) and sequences, in order to enhance the professional expression of such devices.

## 2 Principle of 2D cascaded domain engineering optical phased array

To enable the high-speed and low-power operation of OPA, it is crucial to enhance the efficiency and integration density of dynamic optical components [28]. The capability to dynamically manipulate the phase of photonic channels at gigahertz frequencies is a critically important functionality of integrated electro-optic devices. Lithium niobate (LN) is the most widely used electro-optic material in the industry due to its prominent birefringence, which can be tuned through the linear electro-optic Pockels effect. It satisfies the requirements for exhibiting a broad optical transparency range (0.35–4.5 μm) and high electro-optic activity (31.45 pm/V) [29], making it eligible for use in various applications. Recently, there has been a significant breakthrough in the development of high-quality thin-film LN on insulator (LNOI), which is expected to greatly advance integrated LN photonics [30–32]. However, current research results indicate that the integrated-OPA scheme still faces critical trade-offs in terms of emitter numbers, control electronics, and power consumption [11, 33], [34], [35].

**Figure 1:**
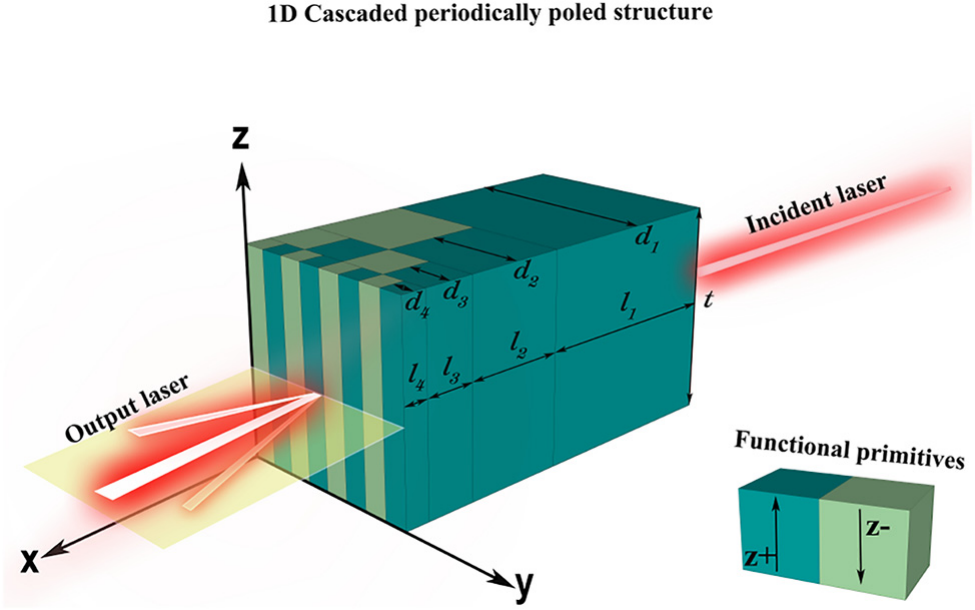
Conceptual image of four-layer cascaded periodically poled electro-optical LiNbO_3_ phased array for one dimension beam steering.

In this study, we demonstrate a feasible method for achieving two-dimensional beam steering utilizing the cascaded periodically poled LiNbO_3_ electro-optical phased array. Firstly, we provide a brief overview of how the cascaded periodically poled LiNbO_3_ electro-optical phased array achieves one-dimensional beam steering. Optical phased arrays are well-known for achieving beam steering through precise control of the relative phase difference (Δ*φ*) between adjacent array elements. The cascaded periodically poled electro-optical LiNbO_3_ phased array is a multilayer structure designed through domain engineering inside the LiNbO_3_ electro-optical crystal, which enables uniform distribution of phase difference between adjacent array elements (i.e. adjacent ferroelectric domains) when the beam exits the crystal. Figure 1 depicts a four-layer cascaded periodically poled electro-optical phased array, from the first layer (input layer) to the last (output layer), where the optical length *l*_*i*_ width *d*_*i*_ of the ferroelectric domains in each layer are halved as the number of cascaded layers increases from the first layer (input layer) to the last one (output layer), following a geometric progression with *l*_1_ = 2*l*_2_ = 4*l*_3_ = 8*l*_4_ and *d*_1_ = 2*d*_2_ = 4*d*_3_ = 8*d*_4_. The electro-optic phase shift induced in a single ferroelectric domain within each layer is also reduced by half. Furthermore, it is noteworthy that the phase shift in positive ferroelectric domains is represented by Δ*φ*_+_ (Δ*φ*_±_ = 2*πl*Δ*n*/*λ*, $\Delta n=-n_{e}^{3}\gamma_{33_{\pm }}E_{z}/2$ ), whereas in negative domains, the sign of Δ*φ*_−_is reversed due to the opposite polarity of Pockels coefficients $\gamma_{33_{\pm }}$ between positive and negative domains of ferroelectric electro-optic crystals. The phase shift of the laser passing through the four-layer cascaded periodically poled electro-optical phased array is presented in Table 1 (Δ*φ*_4_ = 2*πl*_4_Δ*n*/*λ*, $\Delta n=-n_{e}^{3}\gamma_{33}E_{z}/2$ ). The four-layer cascaded periodically poled electro-optical phased array comprises 8 array elements, which correspond to the number of ferroelectric domains in the last cascaded layer. The phase difference between adjacent elements (Δ*φ*) is 2Δ*φ*_4_. The number of array elements doubles with each additional cascaded layer, while the phase difference between adjacent array elements (Δ*φ*) remains constant at 2Δ*φ*_*N*_ (where Δ*φ*_*N*_ represents the phase shift caused by single ferroelectric domain in the last cascaded layer). Based on the theory of optical phased arrays, the deflection angle can be expressed as *θ*_*s*_ = *λ*Δ*φ*/2*πd*, where *λ* and *d* represent the wavelength and width of a single array element respectively. This suggests that the regulation of beam steering can be achieved through Δ*φ*. Therefore, by implementing a single voltage, we can achieve continuous variation of Δ*φ* and realize far-field beam steering.

**Table 1:** The phase shift distributions of four-layer cascaded periodically poled 1-D optical phased array.

Number of cascade layers	Phase shift							
1st layer	+8Δ*φ*_4_							
2nd layer	−4Δ*φ*_4_				+4Δ*φ*_4_			
3rd layer	−2Δ*φ*_4_		+2Δ*φ*_4_		−2Δ*φ*_4_		+2Δ*φ*_4_	
4th layer	−Δ*φ*_4_	+Δ*φ*_4_	−Δ*φ*_4_	+Δ*φ*_4_	−Δ*φ*_4_	+Δ*φ*_4_	−Δ*φ*_4_	+Δ*φ*_4_
Total phase shift	Δ*φ*_4_	3Δ*φ*_4_	5Δ*φ*_4_	7Δ*φ*_4_	9Δ*φ*_4_	11Δ*φ*_4_	13Δ*φ*_4_	15Δ*φ*_4_

**Figure 2:**
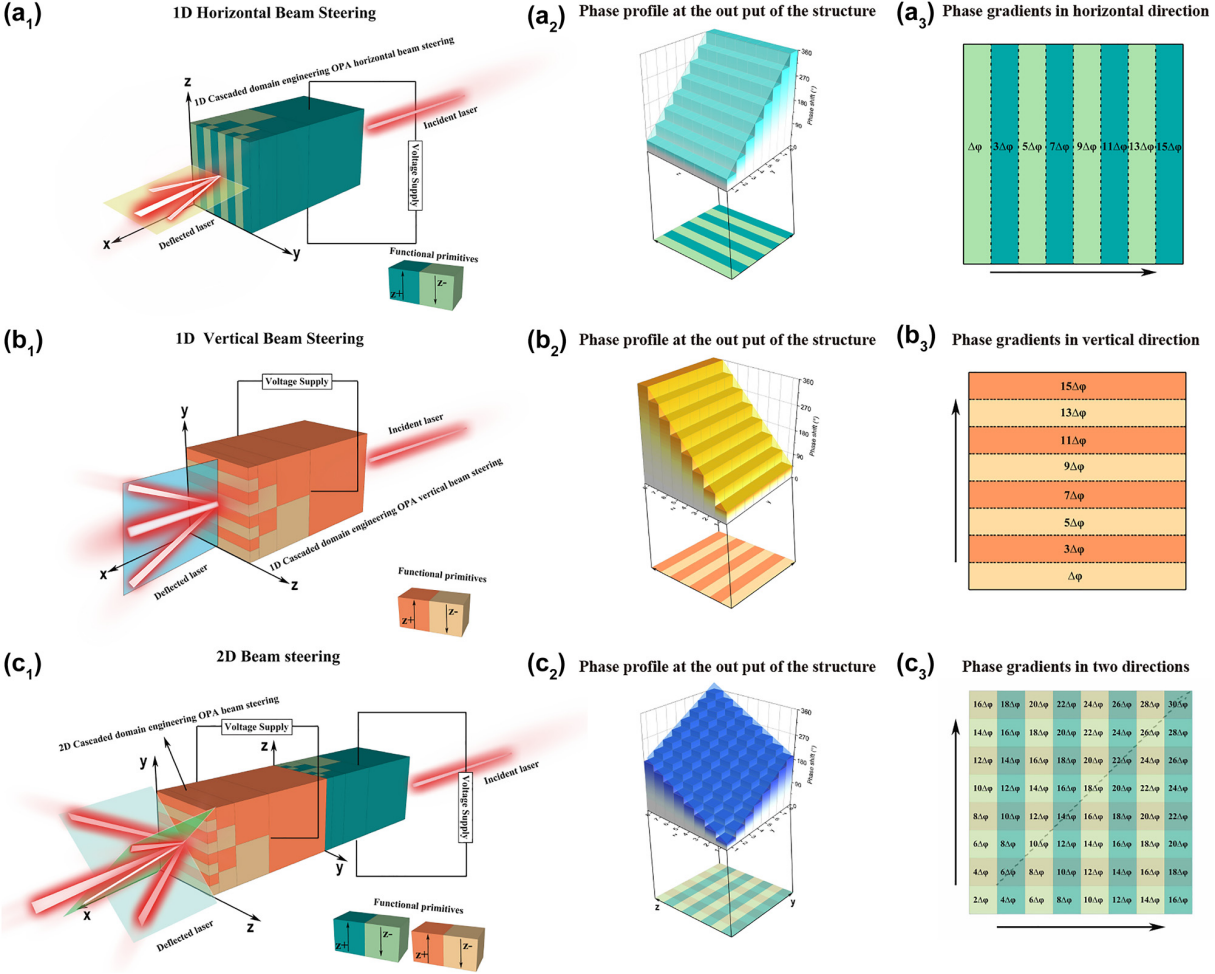
The schematic of cascaded periodically poled electro-optical LiNbO_3_ phased arrays for one dimension and two dimension beam steering. (a_1_) Four-layer cascaded periodically poled electro-optical LiNbO_3_ phased array for one dimension beam steering in *Y* direction with just one electrode; (a_2_) phase profile at the output of the electro-optical phased array for one dimension beam steering in *Y* direction; (a_3_) schematic of phase gradients in *Y* direction; (b_1_) four-layer cascaded periodically poled electro-optical LiNbO_3_ phased array for one dimension beam steering in *Z* direction with just one electrode; (b_2_) phase profile at the output of the electro-optical phased array for one dimension beam steering in *z* direction; (b_3_) schematic of phase gradients in *z* direction; (c_1_) double four-layer cascaded periodically poled electro-optical LiNbO_3_ phased array for two dimension beam steering by applying a pair of electrodes; (c_2_) phase profile at the output of the electro-optical phased array for two dimension beam steering; (c_3_) schematic of phase gradients in *Y* and *Z* direction.

An electro-optical phased array consisting of four cascaded periodically poled LiNbO_3_ layers propagating in the *X* direction is depicted in Figure 2a_1_, which can achieve horizontal beam steering with only one electric field applied from the *Z* direction in LiNbO_3_. The angle of beam steering is controlled by the horizontal phase gradient (as shown in Figure 2a_2_), while the distribution of phase gradient with a 2Δ*φ*_4_ phase difference between adjacent domains is illustrated in Figure 2a_3_. We can achieve continuous horizontal beam deflection by manipulating the electric field. Then, the four-layer cascaded periodically poled LiNbO_3_ electro-optical phased array was rotated by 90° around the *X*-axis, as depicted in Figure 2b_1_. In this case, the electric field applied from the *Z* direction in LiNbO_3_ causes a change in the direction of phase gradient from horizontal to vertical (Figure 2b_2_), resulting a 2Δ*φ*_4_ phase difference between adjacent domains (Figure 2b_3_) and subsequently altering the direction of beam deflection. The above two schemes achieve horizontal and vertical beam steering, respectively, by constructing the corresponding horizontal and vertical phase gradients. For optimal two-dimensional beam steering of an optical phased array, a phase gradient must be present along any arbitrary direction within the plane to achieve beam steering in that particular direction. The configuration of the two-dimensional beam steering system is illustrated in Figure 2c_1_, comprising a pair of four-layer cascaded periodically poled LiNbO_3_ electro-optical phased array, with one array rotated by 90° around the *X*-axis relative to the other. The beam first passes through a structure capable of generating a horizontal phase gradient, and then enters another structure capable of producing a vertical phase gradient. By manipulating the two independent electric fields applied to these structures, the plane of the phase gradient can be tilted in any direction when the beam exits the entire system. In this case, the phase distributions of the light at the exit surface of the structure are divided into 8 × 8 regions, which is equivalent to a two-dimensional optical phased array with 8 × 8 array elements. We assume that the phase difference between two adjacent array elements (ferroelectric domains) in both horizontal and vertical directions is equal after applying electric fields. Furthermore, these two phase gradients increase toward the positive direction in both directions as illustrated in Figure 2a_3_ and b_3_. After modulating the phases of these two structures, the resulting light’s phase distribution at the exit surface is shown in Figure 2c_3_. It is evident that the phase gradient increases diagonally with a phase difference of 4Δ*φ*_4_.

The present study demonstrates two-dimensional beam steering based on two cascaded one-dimensional periodically poled structures on lithium niobate. Specifically, the electro-optically induced phase shift is determined by an electric field applied in the *Z* direction of LiNbO_3_. It should be noted that under such an electric field, the index ellipsoid of LiNbO_3_ can be described by Eq. (1)

(1) $$\begin{array}{ll} & \left(\frac{1}{n_{o}}+\gamma_{13}E_{z}\right)x^{2}+\left(\frac{1}{n_{o}}+\gamma_{13}E_{z}\right)y^{2}+\left(\frac{1}{n_{e}}+\gamma_{33}E_{z}\right)z^{2}=1, \end{array}$$

the refractive indices along the *X*, *Y*, and *Z* axes after the application of an electric field are presented below

(2) $$\left\{\begin{array}{c} n_{x}=n_{y}=n_{o}-\frac{1}{2}\gamma_{13}n_{o}^{3}E_{z}\\ n_{z}=n_{e}-\frac{1}{2}\gamma_{33}n_{e}^{3}E_{z} \end{array}\right..$$

The incident laser beam, with its polarization direction parallel to the *Z* axis, interacts with the first cascaded periodically poled structure. The electro-optically induced phase shift in this structure is determined by Δ*φ*_*z*_ = 2*πl*Δ*n*/*λ*, $\Delta n_{z}=-n_{e}^{3}\gamma_{33}E_{z1}/2$ . However, upon entering the second cascaded periodically poled structure, the polarization direction parallel to the *Y* axis and electro-optically induced phase shift are determined by Δ*φ*_*y*_ = 2*πl*Δ*n*_*y*_/*λ*, where $\Delta n_{y}=-n_{o}^{3}\gamma_{13}E_{z2}/2$ . Two independent voltages *E*_*z*1_ and *E*_*z*2_ suffice for two-dimensional beam steering in the proposed structure.

## 3 Theory between 2D optical phased arrays and cascaded domain engineering sequences

**Figure 3:**
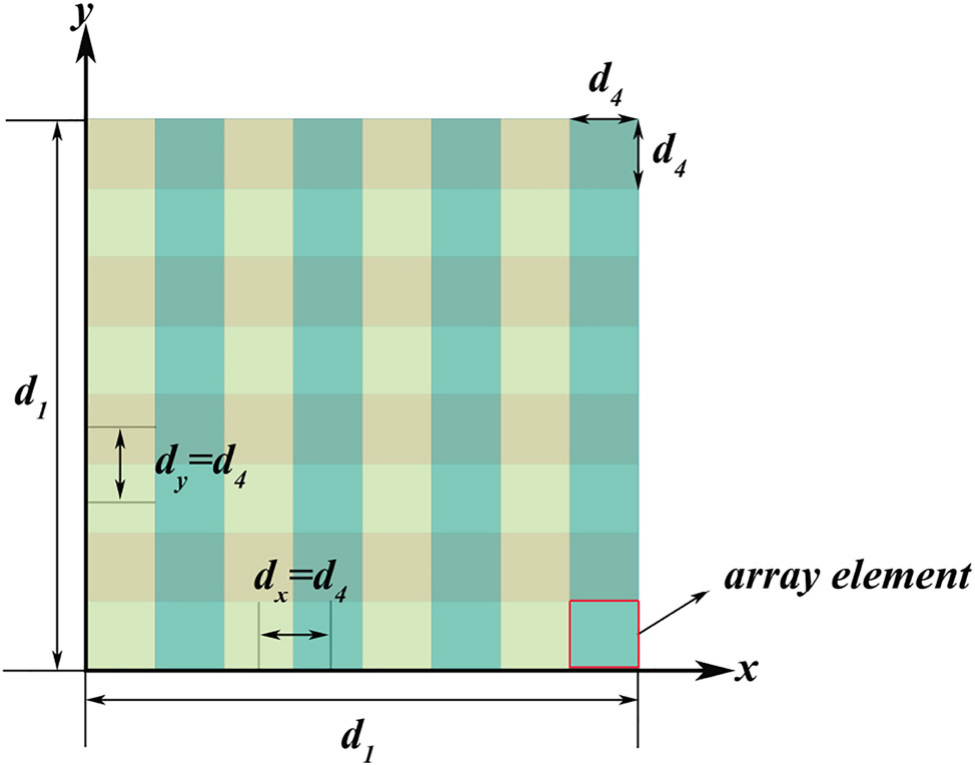
The end face when the laser beam exiting the double four-layer cascaded periodically electro-optical LiNbO_3_ phased arrays forms an 8 × 8 plane optical phased arrays.

The end face of the LiNbO_3_ crystal forms an optical phased array in a plane of *N*_*x*_ × *N*_*y*_ when the laser beam exits. Figure 3 shows the end face when the laser beam exiting the two four-layer cascaded periodically poled LiNbO_3_ electro-optical phased array, the end face forms an 8 × 8 optical phased arrays with array elements spaced *d*_*x*_ = *d*_4_ and *d*_*y*_ = *d*_4_ respectively. The far-field *E* at a certain point *r* in space, can be expressed as the mathematical sum of all array elements’ radiation patterns *F*_*xy*_(*θ*_*x*_, *θ*_*y*_) with appropriate phase factors

(3) $$E\left(r\right)=\overset{N_{x}-1}{\underset{n_{x}=0}{\sum }}\overset{N_{y}-1}{\underset{n_{y}=0}{\sum }}A_{xy}{\text{e}}^{\text{j}\beta_{xy}}F_{xy}\left(\theta_{x},\theta_{y}\right)\frac{{\text{e}}^{-\text{j}k_{0}\left|r-s_{xy}\right|}}{r-s_{xy}},$$

where *A*_*xy*_ is the field amplitude and *β*_*xy*_ is the relative phase at each element. *F*_*xy*_(*θ*_*x*_, *θ*_*y*_) represents the far-field of each element with respect to its own phase center (hence the index *xy*), *k*_0_ is the free-space wave vector and *s*_*xy*_ is the position of array element. Some further simplifications are possible. *F*_*xy*_(*θ*_*x*_, *θ*_*y*_) can be regarded as being referenced to the same phase center *F*_*xy*_(*θ*_*x*_, *θ*_*y*_) ≃ *F*_00_(*θ*_*x*_, *θ*_*y*_) = *F*(*θ*_*x*_, *θ*_*y*_). The denominator in Eq. (3) can be approximated by $\left|r-s_{xy}\right|≃\left|r\right|=R$ . A more precise approximation is required for the phase factor, as it is highly sensitive to variations $\left|r-s_{xy}\right|≃R-u_{r}\cdot s_{xy}$ , where *u*_*r*_ represents the unit vector in the direction of *r*. The simplified result is as follows

(4) $$E\left(r\right)=\underset{1}{\underset{︸}{F\left(\theta_{x},\theta_{y}\right)\frac{{\text{e}}^{-\text{j}k_{0}R}}{R}}}\underset{2}{\underset{︸}{\overset{N_{x}-1}{\underset{n_{x}=0}{\sum }}\overset{N_{y}-1}{\underset{n_{y}=0}{\sum }}A_{xy}{\text{e}}^{\text{j}\beta_{xy}}{\text{e}}^{-\text{j}k\cdot s_{xy}}}}.$$

Equation (4) comprises two components. The first component represents the far-field of a single element, while the second component is a summation factor that accounts for all the different contributions, known as the array factor *T*(*θ*_*x*_, *θ*_*y*_)

(5) $$T\left(\theta_{x},\theta_{y}\right)=\overset{N_{x}-1}{\underset{n_{x}=0}{\sum }}\overset{N_{y}-1}{\underset{n_{y}=0}{\sum }}A_{xy}{\text{e}}^{\text{j}\beta_{xy}}{\text{e}}^{-\text{j}k\cdot s_{xy}}.$$

To utilize optical phased arrays as scanning arrays, a fixed phase difference Δ*φ*_*x*,*y*_ must be maintained between the elements: *β*_*xy*_ = *n*_*x*_Δ*φ*_*x*_ + *n*_*y*_Δ*φ*_*y*_, assuming equal amplitude *A*_*xy*_ = *A*. When the elements are uniformly spaced with *s*_*xy*_ = *n*_*x*_ *d*_*x*_ *u*_*x*_ + *n*_*y*_ *d*_*y*_ *u*_*y*_, with *d*_*x*_ and *d*_*x*_ the element spacing in the *x*- and *y*-direction, respectively, Eq. (5) can be written as

(6) $$\begin{array}{ll} T\left(\theta_{x},\theta_{y}\right) & =A{\text{e}}^{\text{j}\gamma }\frac{\sin\left[\frac{N_{x}}{2}\left(k_{0}d_{x}\sin\theta_{x}-\Delta \varphi_{x}\right)\right]}{\sin\left[\frac{1}{2}\left(k_{0}d_{x}\sin\theta_{x}-\Delta \varphi_{x}\right)\right]}\\ & \;\times \frac{\sin\left[\frac{N_{y}}{2}\left(k_{0}d_{y}\sin\theta_{y}-\Delta \varphi_{y}\right)\right]}{\sin\left[\frac{1}{2}\left(k_{0}d_{y}\sin\theta_{y}-\Delta \varphi_{y}\right)\right]} \end{array}$$

with *γ* a similar phase factor as defined above

(7) $$\begin{array}{ll} \gamma & =\frac{N_{x}-1}{2}\left(k_{0}d_{x}\sin\theta_{x}-\Delta \varphi_{x}\right)\\ & \;+\frac{N_{y}-1}{2}\left(k_{0}d_{y}\sin\theta_{y}-\Delta \varphi_{y}\right), \end{array}$$

The maximum of the array factor occurs when both the numerator and denominator approach zero, or alternatively

(8) $$\begin{array}{c} \frac{1}{2}\left(k_{0}d_{x}\sin\theta_{x}-\Delta \varphi_{x}\right)=q\pi \\ \sin\theta_{x}=\left(q+\frac{\Delta \varphi_{x}}{2\pi }\frac{\lambda }{d_{x}}\right), \end{array}$$

with *q* being an integer. A similar expression holds for the *θ*_*y*_ angle. According to Eq. (8), both the main lobe and grating lobe undergo deflection when a uniform phase difference of Δ*φ*_*x*_ is applied. The deflection angle of the main lobe can be determined by

(9) $$\theta_{x,\text{main}}=\sin^{-1}\left(\frac{\Delta \varphi_{x}}{2\pi }\frac{\lambda }{d_{x}}\right).$$

It is obvious that the control of two-dimensional beam steering can be achieved through Δ*φ*_*x*_ and Δ*φ*_*y*_. Therefore, we can conclude that the structure for two-dimensional beam steering consists of two *N*-layer cascaded periodically poled LiNbO_3_ electro-optical phased array (*N* represents the number of cascaded layer). The values of Δ*φ*_*x*_ and Δ*φ*_*y*_ can be described as follows

(10) $$\begin{array}{c} \Delta \varphi_{x}=2\Delta \varphi_{N}=-2\pi l_{N}n_{e}^{3}\gamma_{33}E_{z}/\lambda ,\\ \Delta \varphi_{y}=2\Delta \varphi_{N}^{\prime }=-2\pi l_{N}^{\prime }n_{o}^{3}\gamma_{13}E_{z}^{\prime }/\lambda , \end{array}$$

where Δ*φ*_*N*_ and $\Delta \varphi_{N}^{\prime }$ represent the phase shift caused by single ferroelectric domain in the last cascaded layer of two *N*-layer cascaded periodically poled LiNbO_3_ electro-optical phased array, respectively. *l*_*N*_ and $l_{N}^{\prime }$ represent the length of single ferroelectric domain in the last cascaded layer of two *N*-layer cascaded periodically poled LiNbO_3_ electro-optical phased array, respectively. *γ*_33_ and *γ*_13_ are the Pockels coefficients (*γ*_33_ = 31.45 pm/V, *γ*_13_ = 10.12 pm/V). According to Eq. (9), *θ*_*x*,main_ and *θ*_*y*,main_ are determined by

(11) $$\begin{array}{ll} \theta_{x,\text{main}} & =\sin^{-1}\left(\frac{1}{2\pi }\frac{\lambda }{d_{x}}\frac{-2\pi l_{N}n_{e}^{3}\gamma_{33}E_{z}}{\lambda }\right)\\ & =\sin^{-1}\left(\frac{-l_{N}n_{e}^{3}\gamma_{33}E_{z}}{d_{x}}\right),\\ \theta_{y,\text{main}} & =\sin^{-1}\left(\frac{1}{2\pi }\frac{\lambda }{d_{y}}\frac{-2\pi l_{N}^{\prime }n_{o}^{3}\gamma_{13}E_{z}^{\prime }}{\lambda }\right)\\ & =\sin^{-1}\left(\frac{-l_{N}^{\prime }n_{o}^{3}\gamma_{13}E_{z}^{\prime }}{d_{y}}\right). \end{array}$$

The control of two-dimensional beam steering is achieved through the manipulation of only two independent voltage parameters, *E*_*z*_ and $E_{z}^{\prime }$ , regardless of the number of array elements. Equation (10) demonstrates the impact of cascaded periodically poled sequences on phase control in emitted beams. While Eq. (11) elucidates the underlying physical mechanism between these sequences and the scanning capability of optical phased arrays.

## 4 Two-dimensional beam steering

**Figure 4:**
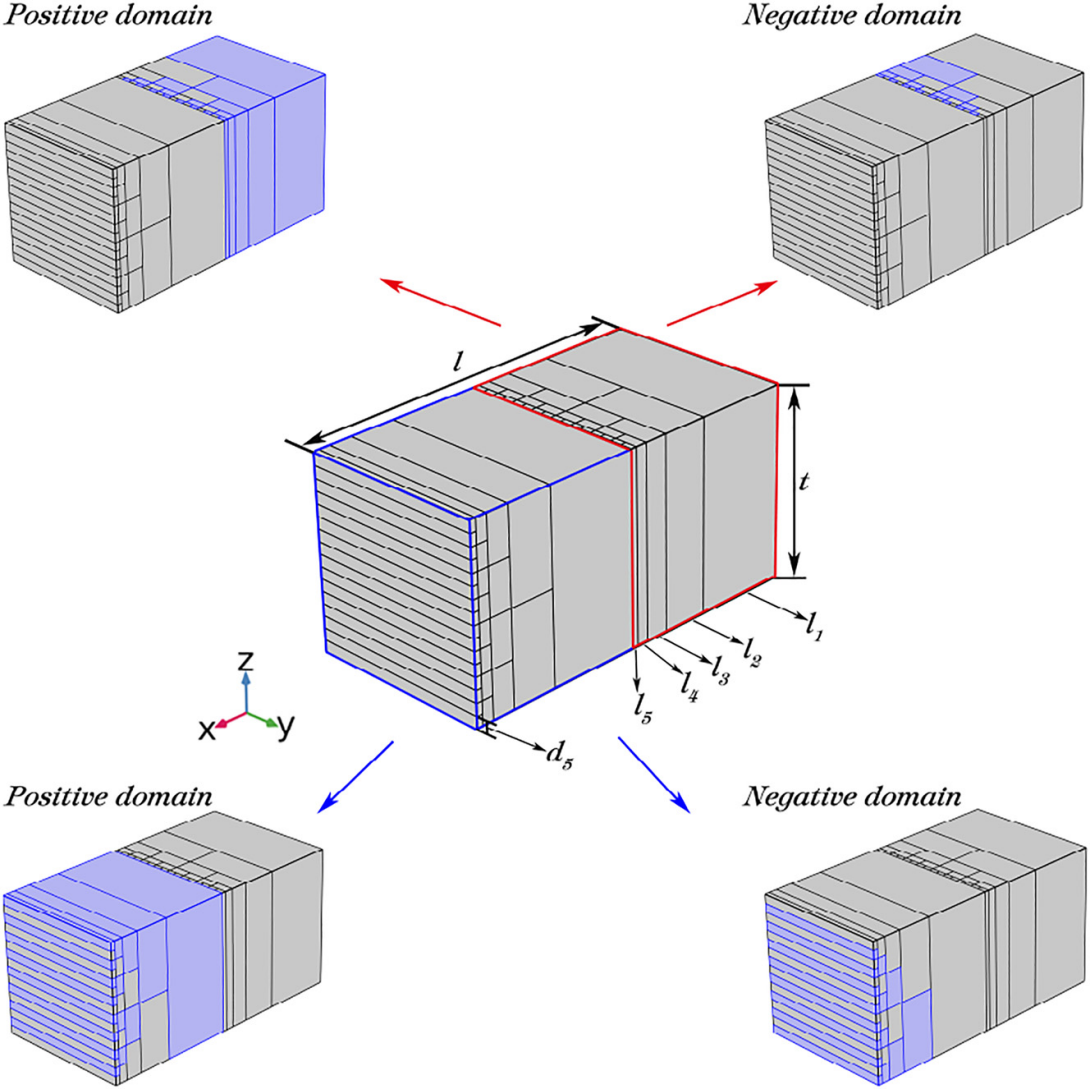
The overall layout simulated model of the two-dimensional electro-optical phased array with the elements number of 16 × 16 which consists of two five-layer cascaded periodically poled LiNbO_3_ and the arrangement of positive and negative ferroelectric domains.

In the theoretical analysis presented in the previous section, two four-layer cascaded periodically poled LiNbO_3_ optical phased array with an element number of 8 × 8 were used to illustrate the beam steering principle. In this section, we aim to better demonstrate the beam steering ability of this structure by enlarging the overall layout of the two-dimensional electro-optical phased array to 16 × 16 array elements, as shown in Figure 4. This array comprises two cascaded periodically poled LiNbO_3_ structures, each with five cascaded layers. The arrangement of positive and negative ferroelectric domains is illustrated in Figure 4, where the lengths of the ferroelectric domains from the first to last cascaded layer are *l*_1_ = 8000 μm, *l*_2_ = 4000 μm, *l*_3_ = 2000 μm, *l*_4_ = 1000 μm and *l*_5_ = 500 μm. Additionally, the width of a single ferroelectric domain in the last cascaded layer is *d*_5_ = 1 μm (*d*_*x*_ = *d*_*y*_ = *d*_5_).

**Figure 5:**
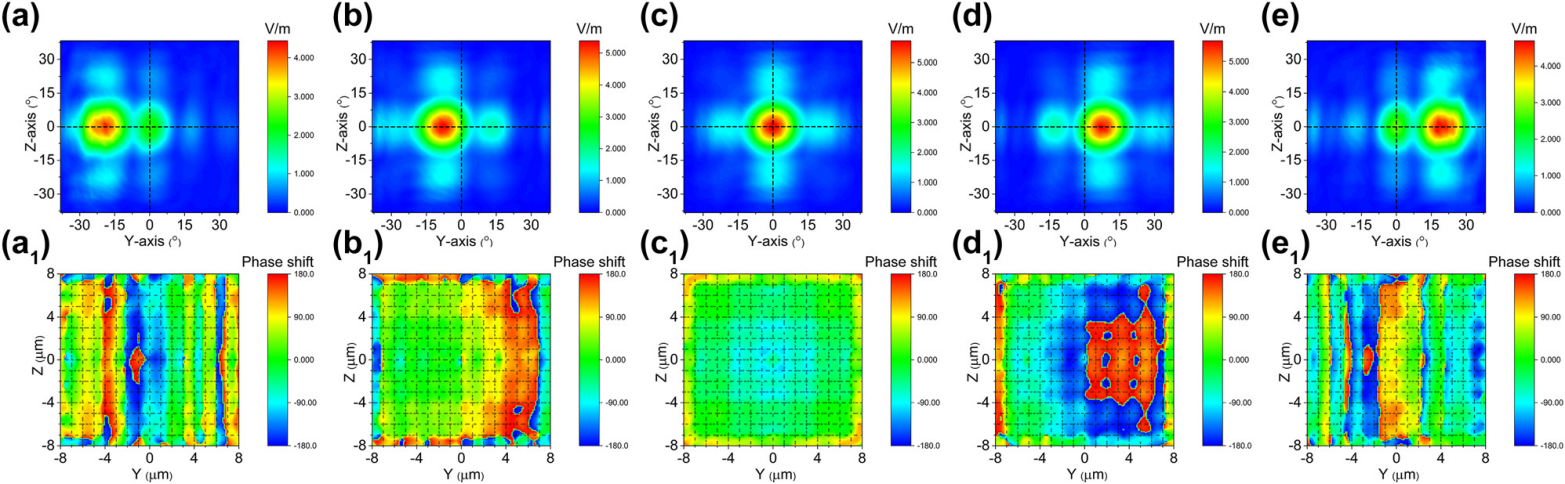
Cascaded domain engineering optical phased array one-dimensional beam steering along the *y *axis. (a–e) The simulated beam steering along *y* axis by only applying voltage *E*_*z*_ (Multimedia view), (a_1_–e_1_) and the phase distributions at the end face of two-dimensional electro-optical phased array (Multimedia view).

We utilized the afore mentioned model to conduct a simulation of the two-dimensional electro-optical phased array’s beam steering capability. The length of the model used in simulation has been simplified and compensated by increasing the applied voltage to reduce computational effort. However, this simplification does not affect the actual beam deflection ability of the two-dimensional electro-optical phased array. Figure 5a–e depict the outcomes of beam steering along the *y* solely through application of electric field *E*_*z*_, which generates a phase gradient exclusively in the *y* direction. The deflected angle from Figure 5a–e (Multimedia view) correspond to applied voltages of are −64 V (−4 V/μm), −25.6 V (−1.6 V/μm), 0 V (0 V/μm), 25.6 V (1.6 V/μm) and 64 V (4 V/μm) with values of −20.225°, −8.837°, 0°, 8.104° and 20.38° respectively. Figure 5a_1_–e_1_ (Multimedia view) illustrate the phase distributions at the end face of a two-dimensional electro-optical phased array corresponding to the deflection angle. The phase differences Δ*φ*_*y*_, calculated using Eq. (10), are 0.233*π*, 0.0934*π*, 0*π*, −0.0934*π* and −0.233*π* with the applied voltage *E*_*z*_ respectively, agree with Figure 5a_1_–e_1_.

**Figure 6: j_nanoph-2023-0382_fig_006:**
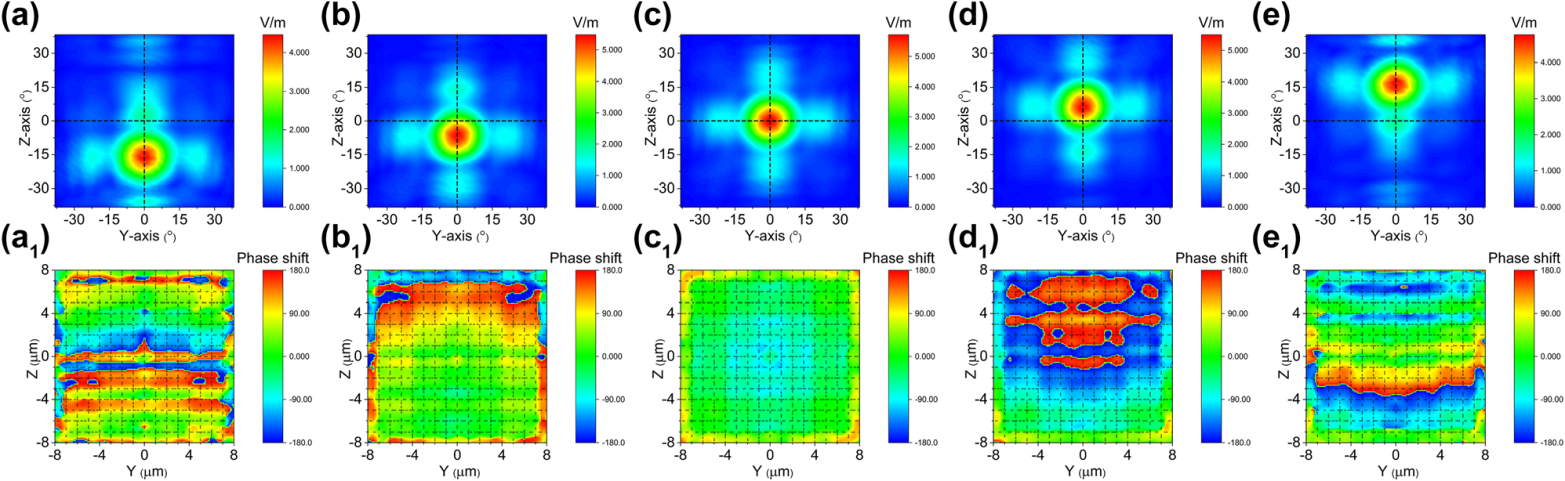
Cascaded domain engineering optical phased array one-dimensional beam steering along the *z *axis. (a–e) The simulated beam steering along *z* axis by only applying voltage $E_{z}^{\prime }$ (Multimedia view), (a_1_–e_1_) and the phase distributions at the end face of two-dimensional electro-optical phased array (Multimedia view).

Similarly, the direction of beam steering along the *z* can be achieved solely by electric field $E_{z}^{\prime }$ , as illustrated in Figure 6a–e (Multimedia view). According to Eq. (10), the Pockels coefficient *γ*_13_ is utilized for inducing electro-optical phase shift in the *z* direction. Consequently, electric field $E_{z}^{\prime }$ exceeds *E*_*z*_ for a given deflection angle. The beam steering along the *Z* direction with drive voltages of −160 V (−10 V/μm), −64 V (−4 V/μm), 0 V (0 V/μm), 64 V (4 V/μm) and 160 V (10 V/μm) are depicted in Figure 6a–e. The deflected angles in Figure 6a–e are −16.355°, −6.882°, 0°, 6.667° and 16.306° with the electro-optically introduced phase differences Δ*φ*_*z*_ of 0.1914*π*, 0.0766*π*, 0*π*, −0.0766*π* and −0.1914*π* as shown in Figure 9 a_1_–e_1_ (Multimedia view). It is evident that the phase gradients increase along the *Z* direction for Figure 6a_1_ and b_1_ while they decrease for Figure 6d_1_ and e_1_.

**Figure 7:**
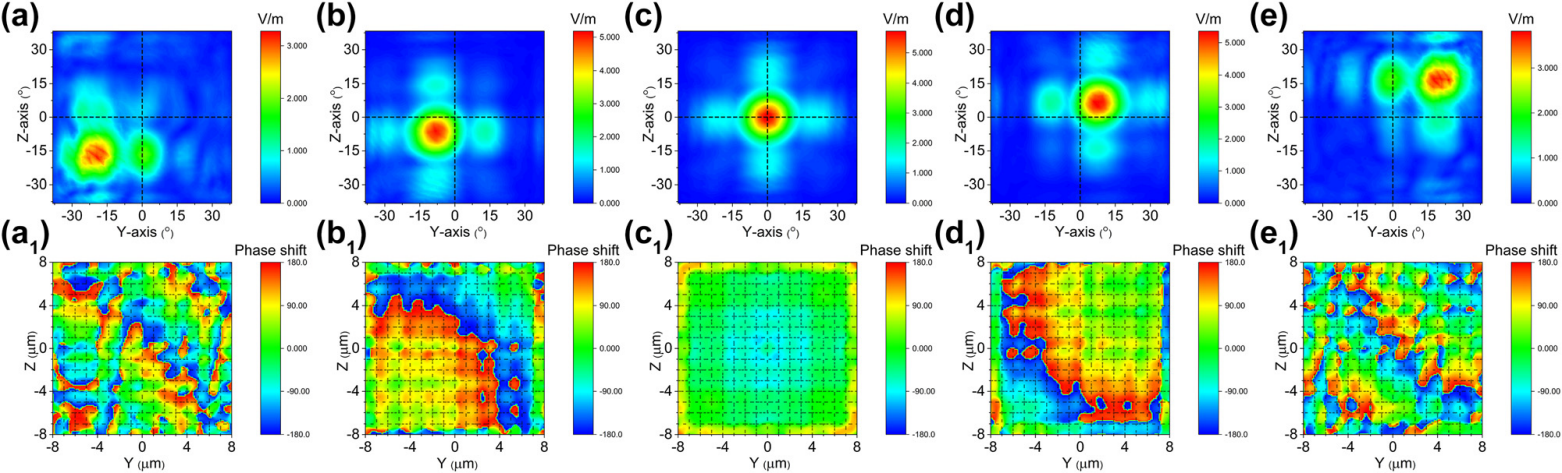
Cascaded domain engineering optical phased array two-dimensional beam steering along plannar diagonal direction. (a–e) The simulated beam steering along planar diagonal direction by simultaneously applying voltage *E*_*z*_ and $E_{z}^{\prime }$ (Multimedia view), (a_1_–e_1_) and the phase distributions at the end face of two-dimensional electro-optical phased array (Multimedia view).

Simultaneous application of *E*_*z*_ and $E_{z}^{\prime }$ enables two-dimensional beam steering, controlling the horizontal and vertical direction angles respectively. Planar diagonal beam steering can be achieved (as shown in Figure 7a–e) through linearly varying *E*_*z*_ and $E_{z}^{\prime }$ , with maximum drive voltages of ±64 V (±4 V/μm) and ±160 V (±10 V/μm) for *E*_*z*_ and $E_{z}^{\prime }$ respectively. At zero bias of both *E*_*z*_ and $E_{z}^{\prime }$ , the beam is positioned at its coordinate home position without deflection. However, when the bias signals are applied to *E*_*z*_ and $E_{z}^{\prime }$ , beam steering occurs. The direction of steering is determined by the polarity of the applied voltage or sign of introduced phase shift. The maximum drive voltages result in an electro-optic phase shift of Δ*φ*_*y*_ = ±0.1914*π* and Δ*φ*_*z*_ = ±0.233*π*, thereby limiting the corresponding steering range to *θ*_*y*_ = ±20° and *θ*_*z*_ = ±16°. Figure 7a–e (Multimedia view) illustrate the deflection positions of the beam when applying five sets of voltages (−64 V (−4 V/μm) and −160 V (−10 V/μm), −25.6 V (−1.6 V/μm) and −64 V (−4 V/μm), 0 V (0 V/μm) and 0 V (0 V/μm), 25.6 V (1.6 V/μm) and 64 V (4 V/μm), 64 V (4 V/μm) and 160 V (10 V/μm)) to *E*_*z*_ and $E_{z}^{\prime }$ . Figure 7 a–e (Multimedia view) illustrate the phase distributions at the end face of a two-dimensional electro-optical phased array. It is evident that the phase gradient varies diagonally with a magnitude equivalent to Δ*φ*_*y*_ + Δ*φ*_*z*_. The phase profile has been designed with a desirable gradient to enable continuous beam steering, as demonstrated in the supplementary materials.

**Figure 8:**
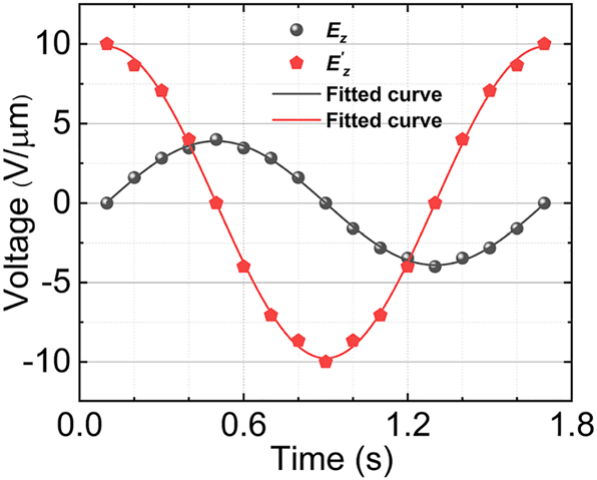
The applied sinusoidal voltages of *E*_*z*_ and $E_{z}^{\prime }$ respectively.

**Figure 9:**
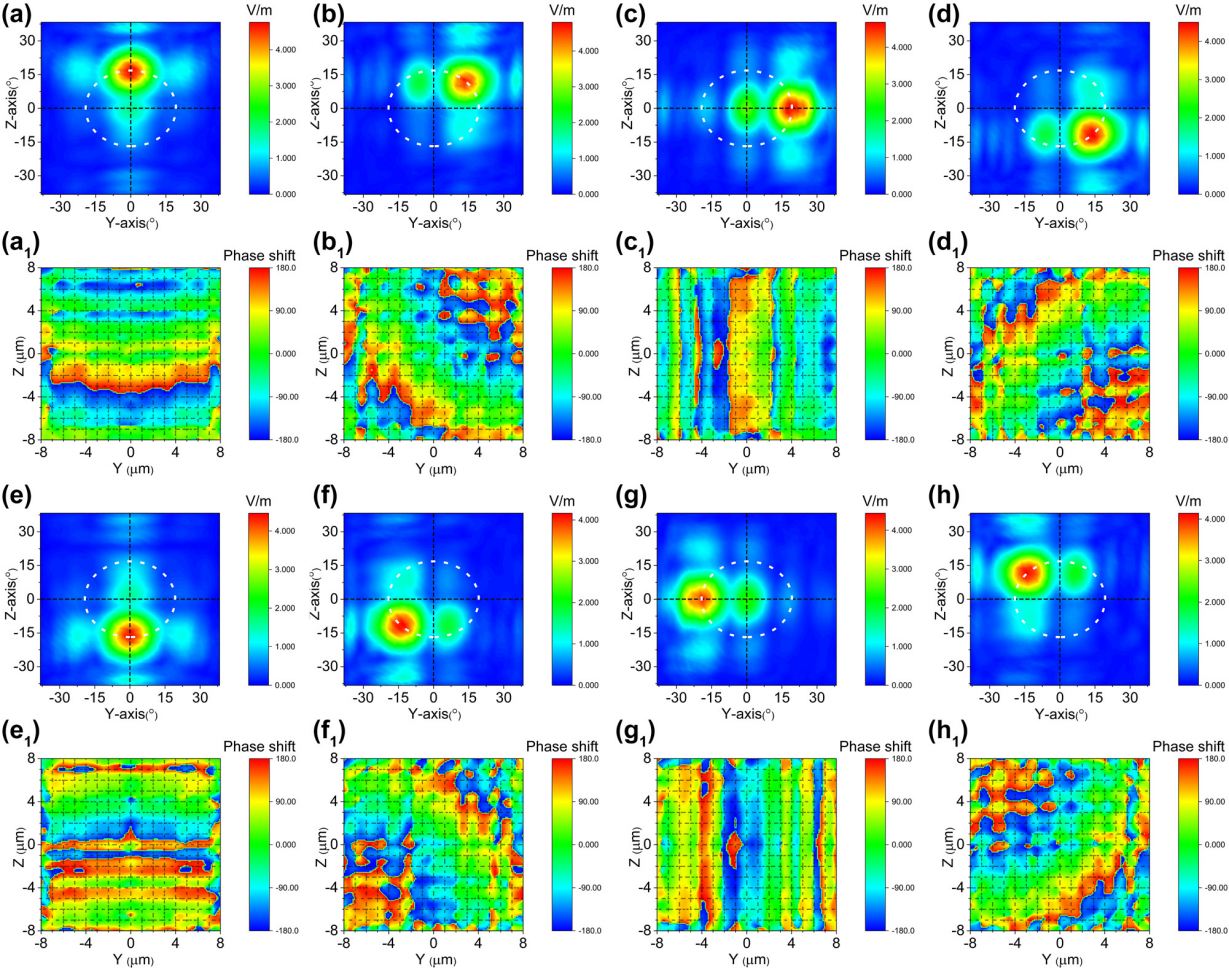
Cascaded domain engineering optical phased array two-dimensional beam steering with the steering path resembles a circle. (a–h) The simulated two dimensional beam steering with the path resembles a circle (Multimedia view), and (a_1_–h_1_) the phase distributions at the end face of two-dimensional electro-optical phased array corresponding to the steering angle (Multimedia view).

As depicted in Figure 7a–h, the trajectory of beam steering is governed by two independent *E*_*z*_ and $E_{z}^{\prime }$ electric fields, indicating that the trajectory can be programmed through these two electric fields. When electric fields are applied to *E*_*z*_ and $E_{z}^{\prime }$ respectively as illustrated in Figure 8, with identical frequency and phase difference of *π*/2, the amplitude of $E_{z}^{\prime }$ is three times that of *E*_*z*_. The beam steering trajectory is illustrated in Figure 9a–e (Multimedia view), and the corresponding phase distributions at the end face are presented in Figure 9 a_1_–h_1_ (Multimedia view). Notably, the direction of phase gradient change aligns precisely with that of beam steering. It is evident that the steering trajectory exhibits circular characteristics, resembling one of Lissajous laser scanning patterns. We can programmatically adjust *E*_*z*_ and $E_{z}^{\prime }$ to optimize Lissajous-like laser scanning patterns, thereby improving the efficiency of the scanning process. The scanning speed is a critical factor in determining imaging quality. With its fast response time(on the order of 100 fs or less), LiNbO_3_’s Pockels effect enables two-dimensional electro-optical phased arrays to achieve high-speed communication potential.

Dynamic beam steering processes, as illustrated in Figures 5 [Fig j_nanoph-2023-0382_fig_006]–7 and 9, can be employed to describe the paths of the beam. Specifically, Movie 1 through Movie 4 depict these dynamic processes while Movies 5 through 8 represent the corresponding dynamic phase distributions at the end face of a two-dimensional electro-optical phased array. All dynamic processes are included in supplementary materials.

**Movie 1:** 

**Movie 2:** 

**Movie 3:** 

**Movie 4:** 

**Movie 5:** 

**Movie 6:** 

**Movie 7:** 

**Movie 8:** 

**Figure 10:**
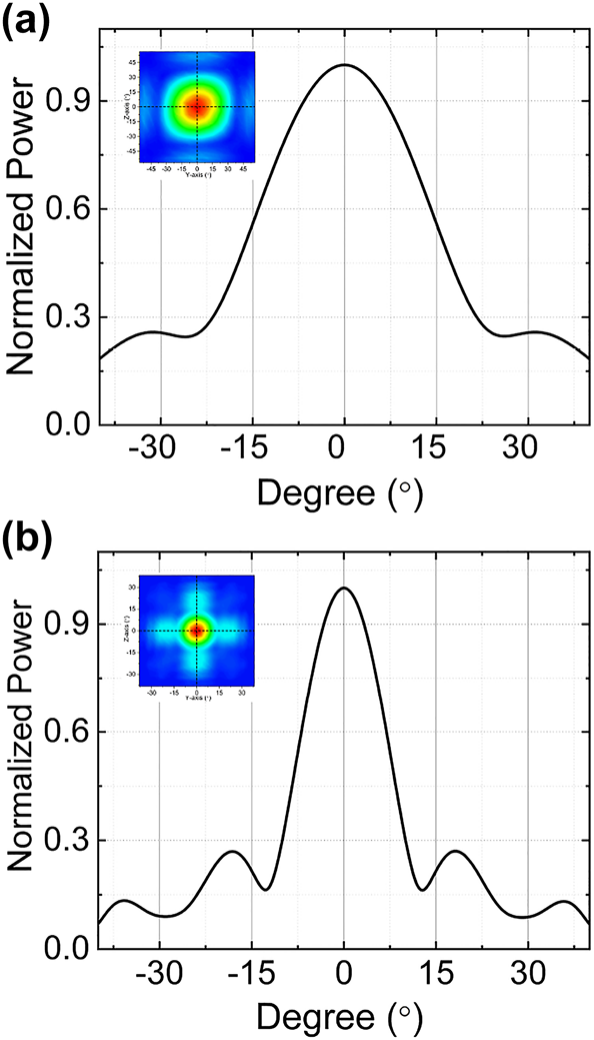
Main beam profiles in the *y* direction and *z* direction with different number of array elements (a) 8 × 8 and (b) 16 × 16.

In most phased arrays, Angular resolution is fundamentally tied to the number of controllable elements in the array. According to the far-field full-width at half-maximum (FWHM) equation of divergence angle: *ψ*_FWHM_ = 0.886⋅*λ*/*Nd*⋅cos *θ*, where *N*, *d* and *θ* represent array number, spacing between array elements and steering angle, respectively. This equation theoretically presents the diffraction limit of the divergence angle of a specific optical aperture *Nd*. The angular resolution is inversely proportional to the total aperture size of the phased array. The trade off between the steering range and angular resolution that can be improved only by increasing the number of control elements. Various types of 2-D scanning on-chip OPAs have been demonstrated, using either matrices of separate antennas where each antenna needs to be individually phase-controlled, or on-chip grating antenna arrays in which wavelength tuning and direct phase modulation steer the beam in two directions separately. However, this conventional design faces challenges when it comes to achieving smaller beam divergence. Achieving a smaller beam divergence necessitates a significant number of phase shifters and emitters, substantially augmenting system complexity and power consumption. In the current two-dimensional electro-optical phased array, the number of array elements is determined solely by the sequence of cascaded periodically poled structures, with each additional sequence doubling the number of elements. All the array elements are controlled by only two units regardless of the number of array elements, which significantly reduces the complexity and power consumption of the control system. The far-field full-width at half-maximum (FWHM) of 8 × 8 array elements (four cascaded layer) and 16 × 16 array elements (five cascaded layer) is shown in Figure 10a and b, where the individual domains in the last layer are both 1 μm. The FWHM divergence angle of 8 × 8 array elements (four cascaded layer) is 32° and the FWHM divergence angle of 16 × 16 array elements (four cascaded layer) is 16°. The beam divergence is reduced with the number of array elements increases from 8 × 8 to 16 × 16. Both the 32° and 16° divergence angles appear to be quite substantial. However, it is possible to further reduce the divergence angle by increasing the number of cascaded layers, which effectively increases the number of array elements. The advantage of this structure lies in the fact that, regardless of the number of array elements added, only two control units are required. The two-dimensional electro-optical phased array exhibits significant superiority in terms of control units.

From the structural characteristics of the two-dimensional cascaded domain engineering optical phased array, it is evident that the horizontal and vertical beam divergences are determined by the number of cascaded layers of the two cascaded domain engineering optical phased arrays. Increasing the number of cascaded layers leads to a higher number of electric domains in the last cascaded layer, resulting in an increase in the number of array elements and the optical aperture. Consequently, this leads to a reduction in both horizontal and vertical beam divergences. However, enlarging the aperture implies an increase in the thickness of the LN crystals along the direction of applied voltage. Under the condition of the same deflection angle, this necessitates a higher control voltage. Therefore, in the practical fabrication of such a device, an effective trade-off between beam divergence and applied voltage needs to be made. While maintaining beam divergence, it is possible to lower the control voltage by appropriately increasing the overall light transmission length of the entire structure, as indicated by Eq. (11).

## 5 Discussion

The aspect ratio plays a pivotal role in influencing various aspects of the cascade domains engineered optical phased array. It significantly impacts application voltages, deflection angles, and angular resolution. As discerned from Eq. (11), an increase in the aspect ratio of this structure leads to larger deflection angles at the same applied voltage. Conversely, for a fixed angle, a higher aspect ratio necessitates lower voltages. Angular resolution, a crucial performance metric of the optical phased array, is determined by FWHM divergence equation. The angular resolution is inversely proportional to the total aperture size of the phased array. When the total aperture size of the two-dimensional cascaded domain engineering optical phased array remains constant, the aspect ratio does not affect angular resolution. Hence, theoretically speaking, a substantial aspect ratio proves highly advantageous for this architecture.

In our previous research endeavors, we successfully demonstrated the fabrication of a six-layer cascaded domain engineering optical phased array within bulk lithium niobate material [27]. Alongside achieving one-dimensional beam steering, we validated the feasibility of the cascaded domain engineering optical phased array architecture. The bulk six-layer cascaded-domain engineered optical phased array achieved one-dimensional beam scanning at a steering speed of 3 MHz and a steering angle of 1° under an applied voltage of 1000 V. Nevertheless, the advantageous characteristics inherent to this design were not fully harnessed when realized within bulk materials. The limiting factor stemmed from the substantial dimensions of the bulk material, leading to the requirement of elevated driving voltages for optical beam steering. Consequently, this posed constraints on the advantageous attributes related to steering angles and high-speed operation. The original intention behind the design of cascaded domain engineering optical phased array (OPA) was to advance the development of beam steering technology based on electro-optic effects. Previous reports on electro-optic beam steering encountered limitations such as small steering angles, low steering speed and resolution, as well as complex control units. The cascaded domain engineering OPA was proposed to address these challenges. To better tackle the afore mentioned issues, it is essential for cascaded domain engineering OPA to exist in the form of a thin film rather than a bulk crystal, preferably utilizing the LN waveguide. Therefore, for the cascaded domain engineering OPA, the ultimate form of this structure should be realized as a waveguide rather than a bulk LN, maximizing the advantages of the cascaded domain engineering OPA. The recent breakthrough in high-quality thin-film LN on insulator (LNOI), enabling chip scale devices with high modulated speed, low power consumption, and so on, has paved the way for integrated cascaded domain engineering OPAs [30, 36]. Hence, we have initiated efforts towards the realization of a one-dimensional cascaded domain engineering optical phased array on LNOI. Upon the successful implementation of the one-dimensional cascaded-periodic polarization engineered optical phased array on LNOI, the achievement of two-dimensional beam scanning, as illustrated in Figure 2c_1_, becomes feasible.

**Figure 11:**
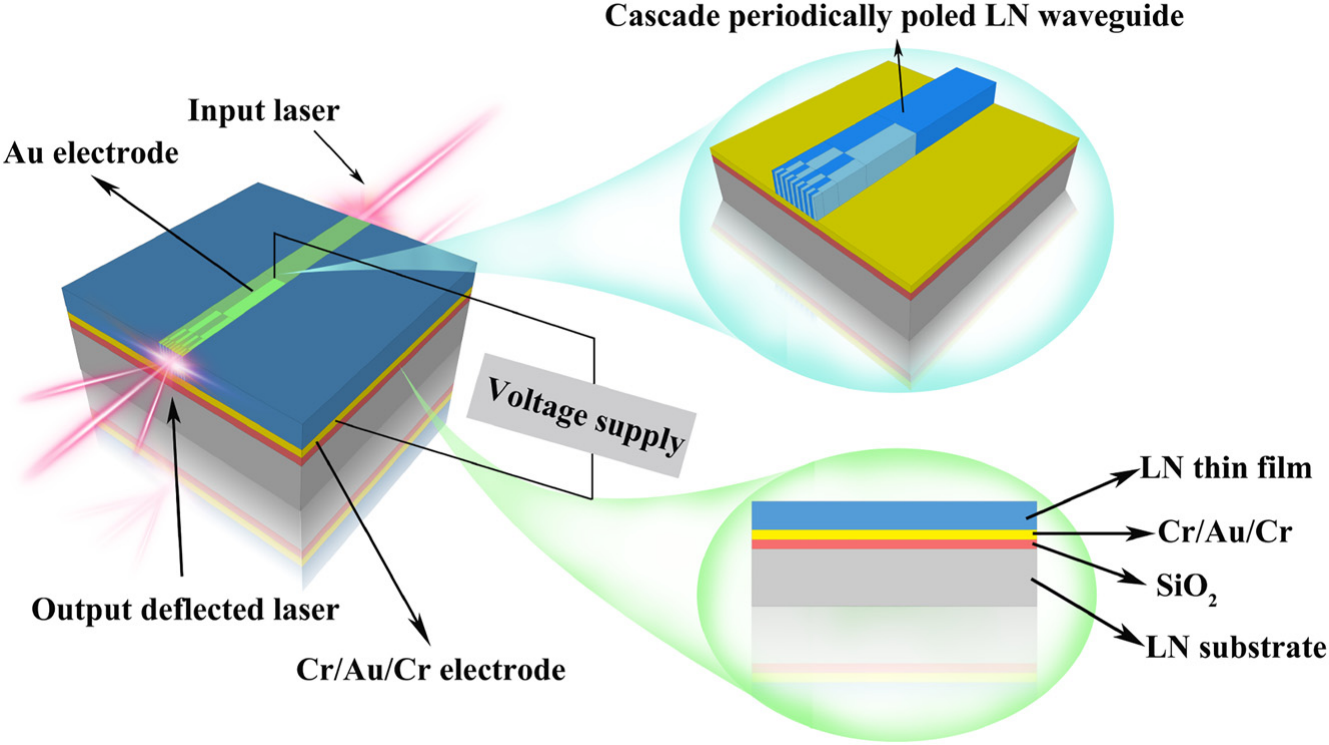
The overall layout of the cascaded domain engineering optical phased array on LNOI.

The schematic illustration of the cascaded domain engineering OPA on LNOI is depicted in Figure 11. The OPA is implemented as a Z-cut on-chip cascaded LN waveguide achieved through domain engineering. This configuration leverages the largest electro-optic effect within the LN crystal and aligns with the externally applied electric field. The cascaded domain engineering OPA LN waveguide is bonded to a SiO_2_-coated LN wafer. Beneath the waveguide, a thick layer of Cu/Au/Cr acts as an electrode, with an additional electrode applied on top of the waveguide. A laser input is coupled into the cascaded domain engineering OPA waveguide, the equal phase difference distribution between each adjacent array element (adjacent domain in the last layer) was constructed by applied the external voltage supply based on the electro-optical effect of LN. Thus, the output wavefront of the light beam can be controlled by the external applied electric field to achieve the beam steering.

**Figure 12:**
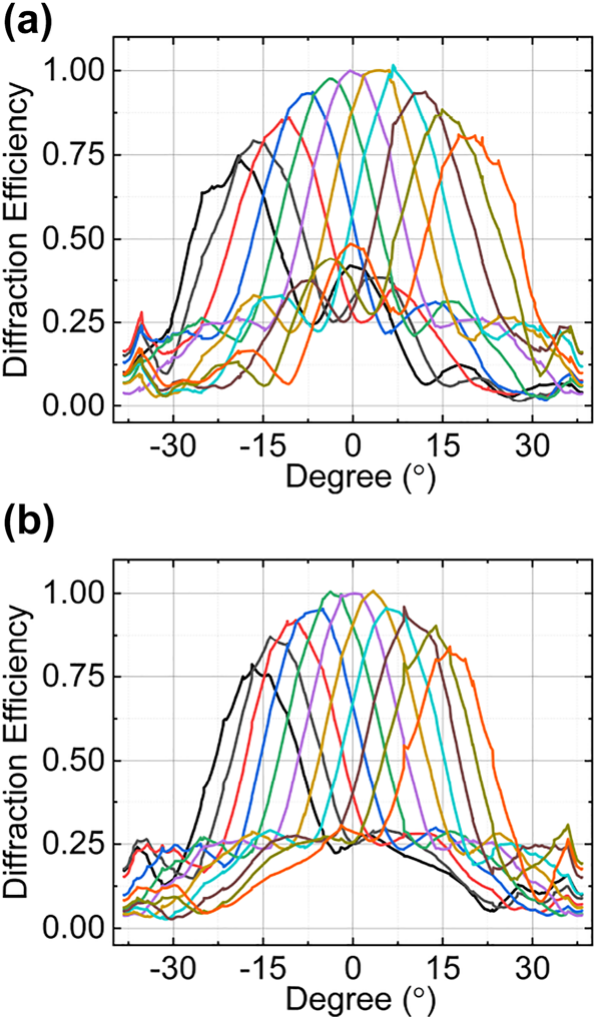
The steering efficiency in different steering direction ((a) along horizontal direction and (b) along vertical direction) versus deflection angle for two-dimensional five-layer cascaded domain engineering OPA.

Another important parameter for OPA is diffraction efficiency, that is, how much of the light input to the device is sent in the desired direction. There are three efficiency considerations for cascaded domain engineering optical phased array. Considering that the ultimate objective of the cascaded domain engineering optical phased array is its implementation within LN waveguides, the primary concern revolves around coupling efficiency, denoting the efficiency of spatial light coupling into the waveguide. For all LNOI devices, the coupling between the LNOI waveguide and the off-chip light sources is one of the keys to reducing the energy consumption of the devices. The most widely used coupling method in LNOI is vertical coupling. Vertical coupling depends on grating couplers (GCs), which are sensitive to polarization and wavelength. In contrast to edge coupling, this coupling scheme is simple to manufacture, has better alignment tolerance, and can also achieve two-dimensional coupling. Over 70 % coupling efficiency for single polarization has been achieved based on GCs. Subsequently, due attention must be given to the inherent losses in LN optical waveguides with cascaded domain engineering structure, which can be analogously compared to domain engineering and periodically poled LNOI (PPLNOI) waveguides. For example, the z-cut PPLN thin-film waveguides with thicknesses of 700 nm and losses as low as 0.7 dB/cm was reported [37, 38], and the high-quality PPLNOI ridge waveguides with losses as low as 0.3 dB/cm were fabricated [37, 39]. Finally, the steering efficiency of the optical beam must also be duly accounted for. The simulation results of the steering efficiency with steering along *y* axis and *z* axis versus the deflection angle are shown in Figure 12(a) and (b), respectively. The steering efficiency is defined as the ratio of the beam intensity during beam steering to that when it is not steering. From Figure 12(a) and (b), it can be observed that whether scanning along the *y* axis (within a range of ±20°) or the *z* axis (within a range of ±16°), the steering efficiency of the beam remains above 75 %.

## 6 Conclusions

In summary, we have proposed a new cascaded domain engineering OPA structure. It could achieve high-speed and continuous beam steering by only one control electronics unit. The beam steering principle behind this structure is the multiple layers cascaded periodic arrangement of ferroelectric domains (positive and negative domains) in the LN crystal. The different Pockels coefficient in the positive and negative ferroelectric domains are used to modulate the phase of the light passing through. Therefore, the light exhibits an equal phase difference distribution when it is emitted from adjacent domains, and the phase difference of light between adjacent domains can be controlled by applying different voltages to achieve far-field beam steering. We have designed a 6-layer on-chip cascaded domain engineering waveguide with steering angle of 4.98° at electric field of 10 V/μm by simulating. The simulation show that the more wide angle could be achieved when the width of single ferroelectric domain further decreasing with a maturing technology. The beam divergences of 0.159° in a steering range of ±5° was achieved with only one phase tuning unit, which means cascaded domain engineering has potential for high-resolution beam steering. Although the device of 6-layer on-chip cascaded domain engineering OPA is not shown in the article, the 6-layer cascaded domain engineering OPA is fabricated in bulk LN and its observed capability of beam steering, with the speed of 3 MHz, is agreed well with simulation results. It is expected that the cascaded domain engineering OPA will achieve the emitter spacing is a half-wavelength or less, with the development of ferroelectric domain poling method. We believe that this new structure provides a new evolution for the realization of highly integrated, high-speed, high-resolution,low-power consumption OPA beam steering systems.
